# Jasmonic Acid-Mediated Aliphatic Glucosinolate Metabolism Is Involved in Clubroot Disease Development in *Brassica napus* L.

**DOI:** 10.3389/fpls.2018.00750

**Published:** 2018-06-04

**Authors:** Li Xu, Huan Yang, Li Ren, Wang Chen, Lijiang Liu, Fan Liu, Lingyi Zeng, Ruibin Yan, Kunrong Chen, Xiaoping Fang

**Affiliations:** Key Laboratory of Biology and Genetic Improvement of Oil Crops, Ministry of Agriculture, Oil Crops Research Institute, Chinese Academy of Agricultural Sciences, Wuhan, China

**Keywords:** *Plasmodiophora brassicae*, clubroot, *Brassica napus*, *Matthiola incana*, glucosinolate, jasmonic acid, MYB28

## Abstract

Glucosinolate (GSL) is associated with clubroot disease, which is caused by the obligate biotrophic protist *Plasmodiophora brassicae*. Due to the complicated composition of GSLs, their exact role in clubroot disease development remains unclear. By investigating clubroot disease resistance in cruciferous plants and characterizing the GSL content in seeds, we can determine if clubroot disease development is related to the components of GSLs. The difference in the infection process between *Matthiola incana* L. (resistant) and *Brassica napus* L. (susceptible) was determined. Root hair infection was definitely observed in both resistant and susceptible hosts, but no infection was observed during the cortical infection stage in resistant roots; this finding was verified by molecular detection of *P. brassicae* via PCR amplification at various times after inoculation. Based on the time course detection of the contents and compositions of GSLs after *P. brassicae* inoculation, susceptible roots exhibited increased accumulation of aliphatic, indolic, and aromatic GSLs in *B. napus*, but only aromatic GSLs were significantly increased in *M. incana*. Gluconapin, which was the main aliphatic GSL in *B. napus* and present only in *B. napus*, was significantly increased during the secondary infection stage. Quantification of the internal jasmonic acid (JA) concentration showed that both resistant and susceptible plants exhibited an enhanced level of JA, particularly in susceptible roots. The exogenous JA treatment induced aliphatic GSLs in *B. napus* and aromatic GSLs in *M. incana*. JA-induced aromatic GSLs may be involved in the defense against *P. brassicae*, whereas aliphatic GSLs induced by JA in *B. napus* likely play a role during the secondary infection stage. Three candidate *MYB28* genes regulate the content of aliphatic GSLs identified in *B. napus*; one such gene was *BnMYB28.1*, which was significantly increased following both the treatment with exogenous JA and *P. brassicae* inoculation. In summary, the increased content of JA during the secondary infection stage may induce the expression of *BnMYB28.1*, which caused the accumulation of aliphatic GSLs in clubroot disease development.

## Introduction

Clubroot disease in cruciferous crops, particularly those belonging to the Brassicaceae family, is caused by the obligate biotrophic protist *Plasmodiophora brassicae* (Dixon, 2009). The life cycle of this pathogen contains the following two distinct phases: the primary phase, which occurs in the root hairs, and the secondary phase, which occurs in the cortical cell of the hypocotyls and roots, resulting in serious disruption of the vascular system and gall formation in the roots of susceptible hosts (Kageyama and Asano, 2009). *Brassica napus* L. is one of the most important oilseed crops worldwide; however, clubroot disease has recently emerged as a serious threat to the production of *B. napus* in China (Ren et al., 2016). Due to the lack of a resistant variety in China, the frequent loss of resistance, and the difficulty in chemical protection, elucidation of the molecular mechanism of clubroot disease is urgently needed (Deora et al., 2012).

The glucosinolates (GSLs), which are synthetized from amino acids and sugars, compose one of the largest known groups of secondary metabolites in the Brassicaceae family (Ishida et al., 2014). The GSLs are classified into three groups (aliphatic, aromatic, and indolic GSL) according to their amino acid precursors (Wittstock and Halkier, 2002). The GSL metabolites have recently attracted scientific interest due to their various beneficial activities. The GSLs not only control pests and have various biological activities related to human health but also play roles in plant defense response against microbial pathogens (Brader et al., 2006; Kusnierczyk et al., 2007; Bednarek et al., 2009; Clay et al., 2009; Liu et al., 2016). Aromatic GSLs are generally considered actors in plant defense against pests, whereas aliphatic GSLs are considered defense compounds against plant pathogens (Ludwig-Muller et al., 1999a; Liu et al., 2016). Indolic GSLs directly or indirectly contribute to clubroot disease development; however, these GSLs are also required for the innate immune response in *Arabidopsis thaliana* (Clay et al., 2009; Ludwig-Muller, 2009). In Brassica cultivars and *A. thaliana* mutants, clubroot disease severity is correlated with the content of indole GSLs, which are considered precursors for auxin biosynthesis (Ludwig-Muller et al., 1999b). High auxin levels are involved in gall formation during the late infection stage, and indolic GSLs are in turn precursors for indole-3-acetic acid (IAA) biosynthesis and are correlated with clubroot disease severity (Ludwig-Muller et al., 1999b; Ludwig-Muller, 2009).

Several plant growth regulators, including auxin, cytokinin, abscisic acid, ethylene, salicylic acid (SA), and jasmonic acid (JA), are modulated during clubroot disease (Devos et al., 2005, 2006; Devos and Prinsen, 2006; Siemens et al., 2006; Knaust and Ludwig-Muller, 2013; Ludwig-Muller, 2014; Lemarie et al., 2015; Lovelock et al., 2016). Published results have mostly focused on investigating the role of IAA involved in gall formation during the late infection stage. In our previous study, IAA acted as a signaling molecule that putatively stimulated root hair infection during the early response of *B. napus* to *P. brassicae* infection (Xu et al., 2016). In addition to IAA, increased cytokinin levels were correlated with clubroot disease symptoms and caused increases in cell division during the beginning of club formation (Siemens et al., 2006). Plasmodia synthesize cytokinin, which induces host cell division during clubroot disease development (Devos et al., 2005). The JA and SA signaling pathways are generally considered antagonistic in disease response (Bari and Jones, 2009). However, SA- and JA-triggered defenses result in resistance after *P. brassicae* inoculation in Bur-0 (partially resistant) and Col-0 (susceptible), respectively (Lemarie et al., 2015). In addition, the SA pathway appears to be more efficient than the JA pathway in clubroot resistance. JA accumulation during clubroot infection has been reported in susceptible Chinese cabbage and Arabidopsis Col-0 and Bur-0 during the secondary infection stage (Grsic et al., 1999; Lemarie et al., 2015).

Transcription factors (TFs) function as components of hormone signaling and modulate plant growth, development and the response to stress (Bari and Jones, 2009). During biotic and abiotic stress responses, GSL biosynthesis is regulated by a complex network of TFs belonging to the R2R3-MYB family, including MYB28, MYB29, and MYB76, which are involved in aliphatic GSL biosynthesis, as well as MYB51, MYB122, and MYB34, which are involved in indolic GSL biosynthesis in Arabidopsis (Li et al., 2013; Frerigmann and Gigolashvili, 2014). Of these TFs, MYB28 plays the most important role in aliphatic GSL biosynthesis, followed by MYB29 and MYB76, which have a partial functional redundancy.

Because the hydrolysis products of GSLs are poisonous, the application of rapeseed as feed is greatly limited (Abbadi and Leckband, 2011). Since the 1980s, rapeseed breeders have sought to obtain low-GSL rape varieties, such as double-low rapeseed with low erucic acid and low GSL in seed (Nesi et al., 2008). Recently, double-low rapeseed plantings have suffered from clubroot disease, which poses a serious threat to the production of *B. napus* in China. Evidence regarding the relationship between the poor resistance of the main rape cultivars and double-low rapeseed is lacking. However, more than 200 types of GSLs exist in the Brassicaceae family (Ishida et al., 2014). The exact role of GSLs in clubroot disease remains controversial, and systematic studies are lacking.

Based on a previous study investigating clubroot disease resistance in Brassicaceae, *M. incana* L. (resistant) and *B. napus* L. (susceptible) were chosen for investigation in this study (Ren et al., 2016). The differences in the infection process of *B. napus* and *M. incana* were determined by performing a solution culture technique. By detecting the content and composition of GSLs at various time points after *P. brassicae* inoculation, the role of GSLs was determined during the response of *B. napus* to *P. brassicae*. These data will provide useful information for elucidating the regulatory mechanism of GSL biosynthesis in clubroot disease.

## Materials and Methods

### Plant Materials and Pathogen Isolates

The *P. brassicae* isolates used in this study were collected from clubroot-infested field plots in Zhijiang, Hubei, China. The resting spores of *P. brassicae* were isolated from the galls using a procedure described by Xu et al. (2016). The resting spores were identified as pathotype 4 according to the differential classification of Williams (1966). The disease status was investigated at 35 days after inoculation (DAI).

The cruciferous plants used in this study were sent for disease resistance identification as previously described (Ren et al., 2016). In total, 14 varieties from seven species were used in this study to detect the GSLs in seeds (**Table 1**). The clubroot-susceptible *B. napus* cultivar ‘zhongshuang 11’ and clubroot-resistant *M. incana* cultivar ‘Francesca’ were used for further study. The crops and ornamentals used in this study were obtained from the Oil Crops Research Institute of the Chinese Academy of Agricultural Sciences or purchased from commercial seed companies.

**Table 1:** Cruciferous plants tested in this study.

	Common name	Botanical name	Source
R1	English wallflower	*Cheiranthus cheiri*	Takii Seed, Kyoto, Japan
R2	Violet orychophragmus	*Orychophragmus violaceus*	Guomei Horticulture Co., Ltd., Zhejiang
R3	Indigowoad root	*Isatidis Radix*	Guomei Horticulture Co., Ltd., Zhejiang
R4	Violet (hot cakes)	*Matthiola incana*	PanAmerican Seed, West Chicago, IL, United States
R5	Violet (Vintage)	*Matthiola incana*	PanAmerican Seed, West Chicago, IL, United States
R6	Violet (Incana)	*Matthiola incana*	PanAmerican Seed, West Chicago, IL, United States
R7	Violet (Francesca)	*Matthiola incana*	PanAmerican Seed, West Chicago, IL, United States
S1	Chinese cabbage (xin 3)	*Brassica rapa pekinensis*	Jingyan Yinong, Beijing
S2	Radish (Banyeweiqing)	*Raphanus sativus*	Takii Seed, Kyoto, Japan
S3	Radish (Baiyudagen)	*Raphanus sativus*	Takii Seed, Kyoto, Japan
S4	Oilseed (zhongshuang 11)	*Brassica napus*	OCRI, CAAS
S5	Oilseed (zhongyou 821)	*Brassica napus*	OCRI, CAAS
S6	Oilseed (BJ003)	*Brassica napus*	OCRI, CAAS
S7	Oilseed (BJ004)	*Brassica napus*	OCRI, CAAS

*OCRI, CAAS: Oil Crops Research Institute of the Chinese Academy of Agricultural Sciences, Wuhan.*

### High-Performance Liquid Chromatography (HPLC) Analysis of GSL Content

Desulfo (DS)-GSLs were extracted according to a procedure previously described by Kim and Ishii (2006) and ISO 9167-1 (1992). The seeds or fresh leaves were ground into powder, and 100 mg of freeze-dried sample was extracted twice in 70% methanol. The crude extracts were loaded on Sephadex A25 columns and desulfated overnight using aryl sulfatase. The 1200 series HPLC system (Agilent Technologies, Santa Clara, CA, United States) equipped with an Inertsil ODS-3 column (GL Science, Tokyo, Japan) was used in the analysis of the GSL content. The external standard method was used for quantitative analysis of individual GSLs, and sinigrin was used as an external standard. The values of the total GSLs were obtained by summing the values of the individually identified GSLs (Kim and Ishii, 2006). Three biological duplicates were performed for each treatment.

### Cytological Observation of Clubroot Disease Development

Clubroot disease development in *B. napus* and *M. incana* was determined using an improved solution culture technique and microscopic investigation (Xu et al., 2016).

The development of root hair infection by *P. brassicae* was investigated at 3, 7, 10, and 14 DAI. The roots were washed with tap water, and a segment (0.5 cm long) was cut from the lateral root during each treatment and time point. The segments were stained with FAA Phloxine B, covered with a coverslip, then observed and imaged under an optical microscope (DMLS, Leica) (Donald et al., 2008).

The development of cortex infection by *P. brassicae* was investigated at 14, 21, and 28 DAI. The roots were washed with tap water, and a segment (0.5 cm long) was cut from the top 0 to 1 cm of the taproot during each treatment and time point. The segments were fixed in FAA for 24 h. After routine fixation and dehydration, the samples were embedded in paraffin and sliced into 8-μm-thick sections. Finally, the sections were observed and imaged under an optical microscope (Jin et al., 2014).

### Extraction and Determination of JA

The infected and control roots were collected at 3, 7, 14, and 28 DAI. A 0.5-ml aliquot of 1-propanol:H_2_O:concentrated HCl (2:1:0.002, v/v/v) was added to 0.1 g of fresh roots, and the mixture was shaken for 30 min at 4°C. After adding 1 ml of dichloromethane, the mixture was shaken for another 30 min and centrifuged at 12,000 × *g* for 5 min. After centrifugation, the bottom organic phase was collected and concentrated with nitrogen. Each sample was redissolved in 1 ml of 80% methanol and extracted using a C18 SPE cartridge (CNWBOND HC-C18, 500 mg, 3 ml). The eluate was evaporated to dryness under vacuum and finally dissolved in 200 μL of methanol: 0.05% formic acid (1:1, v/v). The solution filtered by the filter membrane sterilization was used for quantification of JA. Quantification of JA was performed using our previously published methods (Xu et al., 2016). Three biological duplicates were performed for each treatment.

### Treatment With Exogenous JA

The seedlings were treated with 200 μmol/L of JA (Sigma-Aldrich, St Louis, MO, United States) by adding JA to the soil. The roots of the seedlings were collected 48 h after the treatment for RNA extraction. Three biological duplicates were performed for each treatment.

### Bioinformatic Analysis of the Involvement of BnMYB28 in GSL Biosynthesis

MYB28 TFs related to the GSL biosynthetic pathway have been previously identified in *B. napus, B. rapa, B. oleracea*, and *A. thaliana*, as shown in **Table 2** (Li et al., 2013; Seo et al., 2016; Yin et al., 2017). Multiple alignment analysis of BnMYB28, BrMYB28, BoMYB28 and AtMYB28 was performed using ClustalX2. The phylogenetic relationships of the MYB TFs were analyzed, and a phylogenetic tree was predicted.

**Table 2:** Ortholog list of transcription factors involved in GSL biosynthesis in *B. napus*.

*A.thaliana* (GenBank)		*B. rapa* (Ensembl plants)		*B. oleracea* (Ensembl plants)		*B. napus* (Ensembl plants)	
AtMYB28	AT5G61420	BrMYB28-1	Bra012961	BoMYB28-1	Bo7g098590	BnMYB28-1	BnaA03g40190D
		BrMYB28-2	Bra035929	BoMYB28-2	Bo2g161590	BnMYB28-2	BnaC09g05300D
		BrMYB28-3	Bra029311	BoMYB28-3	Bo9g014610	BnMYB28-3	BnaCnng43220D

### DNA Isolation and PCR Amplification

The infected and control *B. napus* and *M. incana* roots sampled at 3, 7, 14, 21, and 35 DAI were used for DNA isolation and PCR amplification. To avoid contamination by spores adhering to the root surface, the roots were rinsed with tap water. At least five plants were randomly selected for the extraction of the genomic DNA using the CTAB method.

The PCR amplifications were conducted using a pathogen specific primer and the plant reference gene primers listed in Supplementary Table S1. All amplifications were conducted as previously described by Ren et al. (2016).

### RNA Isolation and Real-Time Quantitative PCR (qPCR)

The total RNA was isolated from the control and used to treat *B. napus* and *M. incana* roots at various time points after the inoculation or JA treatment (3, 7, 14, and 28 DAI) using TRIzol Reagent (Invitrogen, Karlsruhe, Germany) according to the manufacturer’s instructions. The Superscript first-strand synthesis system (Invitrogen, Foster City, CA, United States) was used for first-strand cDNA generation. The primers used for the qPCR were designed using Primer Premier 6.0 software and are shown in Supplementary Table S2. The qPCR were performed using our previously published methods (Xu et al., 2016). Three biological duplicates were performed for each treatment.

## Results

### Disease Development Differences Between Resistant and Susceptible Plants

**FIGURE 1:**
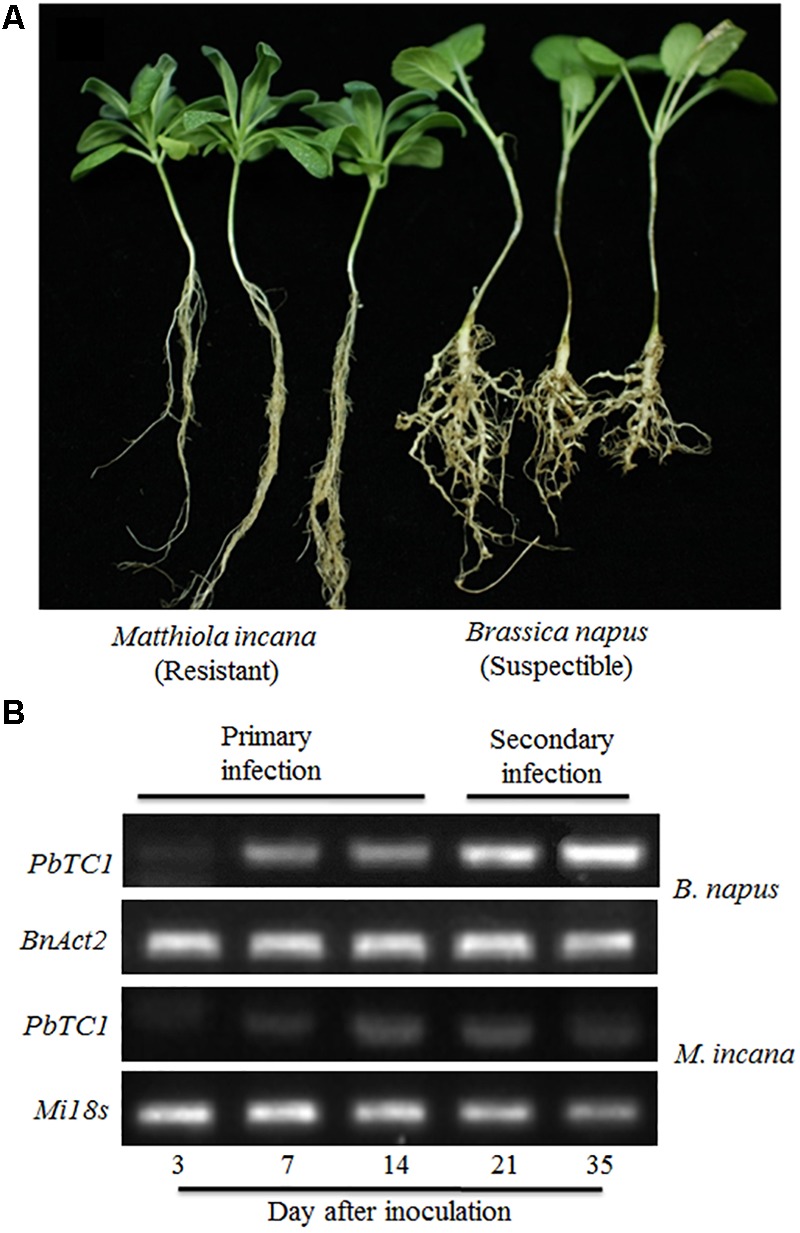
Symptoms of clubroot disease in *Brassica napus* and *Matthiola incana* at 35 days after inoculation (DAI) **(A)** and PCR detection of *P. brassicae* in the roots of *B. napus* and *M. incana* at 3, 7, 14, 21 and 35 DAI **(B)**.

Disease development was evaluated 35 days after the *P. brassicae* inoculation (DAI) of *B. napus* and *M. incana*. The formation of galls was observed on the roots of the *B. napus*, but not *M. incana*, seedlings (**Figure 1A**). *P. brassicae* was detected via PCR analysis at various time points after inoculation. PCR products were obtained from both resistant and susceptible roots (**Figure 1B**). Increased levels of the pathogen were detected in susceptible roots as the disease developed. However, a relatively low level of the pathogen was detected in resistant roots, and the pathogen level decreased after 21 DAI, suggesting that the infection in *M. incana* was at the root hair infection stage.

**FIGURE 2:**
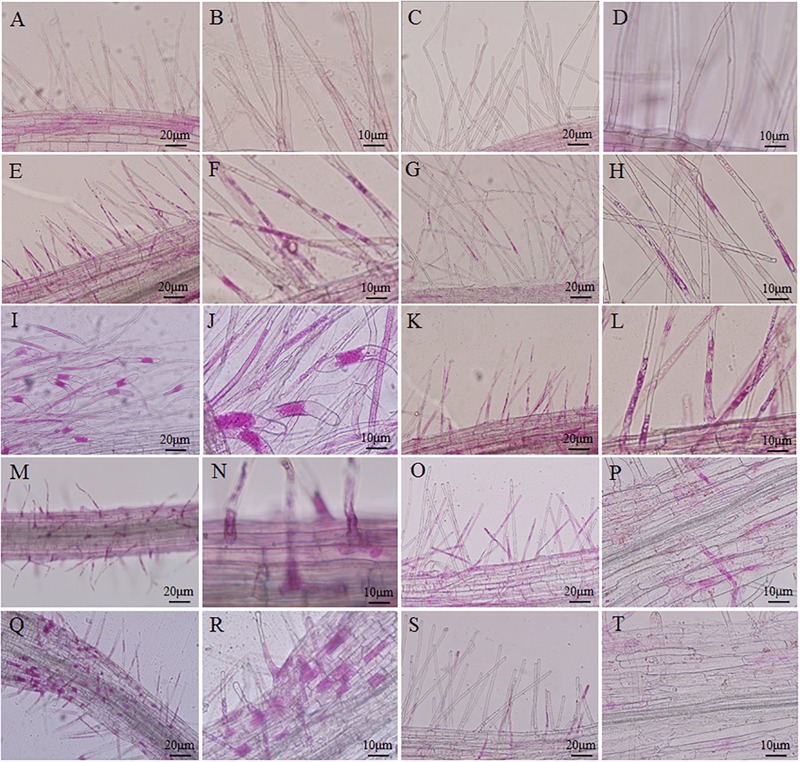
Infection dynamics in the root hairs of resistant and susceptible plants. Root segments were stained with Phloxine B. Staining in the root hairs indicated the presence of root hair infection. **(A,B,E,F,I,J,M,N,Q,R)** Segments of roots of *Brassica napus*. **(C,D,G,H,K,L,O,P,S,T)** Segments of roots of *Matthiola incana*. **(A–D)** Segments of control roots. **(E–H)** Segments of inoculated roots at 3 days after inoculation (DAI). **(I–L)** Segments of inoculated roots at 7 DAI. **(M–P)** Segments of inoculated roots at 10 DAI. **(Q–T)** Segments of inoculated roots at 14 DAI. Adjustments for magnification and illumination were performed to allow optimal viewing of the individual sections.

An improved solution culture technique was employed to investigate the root hair infection process. Phloxine B-stained root hairs were microscopically examined to detect the presence of *P. brassicae* at 3, 7, 10, and 14 DAI in both resistant and susceptible hosts (**Figure 2**). Root hair infection was observed at 3 DAI in both roots (**Figures 2E–H**). Primary plasmodia were visible in the root hairs at 7 DAI, and swollen cells were observed in the root hairs of the susceptible, but not resistant, hosts (**Figures 2I–L**). The zoosporangia formed clusters in the root hairs at this time point, followed by the release of secondary zoospores. The secondary zoospores penetrated the cortical tissues at 10 DAI in the susceptible hosts (**Figures 2M,N**), and the pathogen developed into secondary plasmodia in the cortical cells of the roots at 14 DAI (**Figures 2Q,R**). The secondary infection was initiated by secondary zoospores, which move into the cortical cells to cause secondary infection.

**FIGURE 3:**
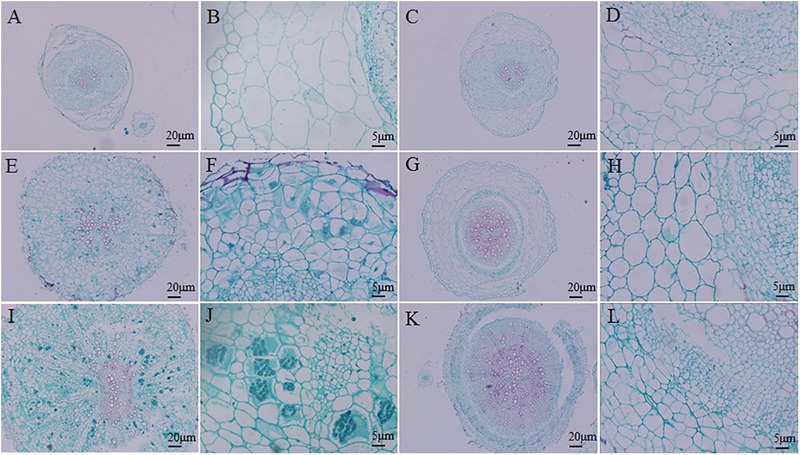
Histocytological analysis of cross-sections of inoculated resistant and susceptible plants. **(A,B,E,F,I,J)** Sections of root of *Brassica napus*. **(C,D,G,H,K,L)** Sections of root of *Matthiola incana.* **(A–D)** Sections of inoculated roots at 14 days after inoculation (DAI). **(E–H)** Sections of inoculated roots at 20 DAI. **(I–L)** Sections of inoculated roots at 28 DAI. Adjustments for magnification and illumination were performed to allow optimal viewing of the individual sections.

A histocytological analysis was performed to investigate cross-sections of the inoculated resistant and susceptible roots at 14, 20, and 28 DAI to uncover dynamic changes in the roots during the cortical infection stage (**Figure 3**). A slight difference was observed between resistant and susceptible hosts at 14 DAI, with the exception that susceptible plants showed secondary plasmodia in the inner cortex (**Figures 3A–D**). However, secondary plasmodia were also observed in the root cortex, and compared with the resistant hosts at 20 DAI, the inoculated susceptible roots showed delayed xylem development and decreased lignified xylem bundles, vessels, and interfascicular fibers were observed (**Figures 3E,F**). The secondary plasmodia proliferated, which caused cellular hypertrophy, division and enlargement of the root cells, and gall formation in the root tissues (**Figures 3I,J**). The secondary plasmodia finally developed into resting spores at 28 DAI in the susceptible roots. Moreover, the root cells were disorderly, and the cell wall thickened, resulting in a serious disruption of the vascular system. In contrast, the resistant roots grew normally during this stage.

In summary, the life cycle of *P. brassicae* was complete in *B. napus*, but not *M. incana*, which did not show any secondary infection as confirmed by PCR.

### GSL in Cruciferous Plant Seeds

**FIGURE 4:**
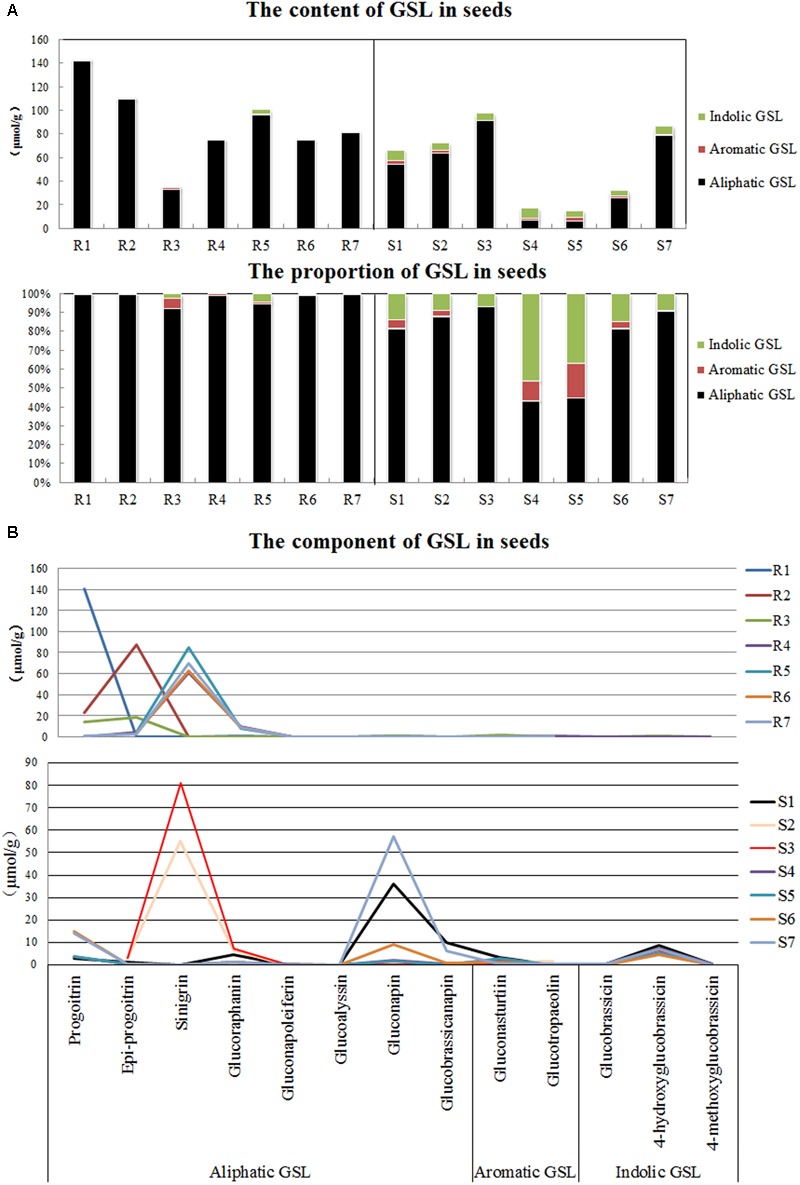
Detection of glucosinolates (GSLs) in seeds of various disease resistance plants. **(A)** Contents of three types of GSLs. **(B)** Proportions of three types of GSLs. R1-R7 refer to the clubroot-resistant plants, and R1-R7 refer to the clubroot-susceptible plants. R1, English wallflower; R2, Violet orychophragmus; R3, Indigowoad Root; R4, Violet (Matthiola hot cakes); R5, Violet (Vintage); R6, Violet (Incana); R7, Violet (Francesca); S1, Chinese cabbage (xin 3); S2, Radish (Banyeweiqing); S3, Radish (Baiyudagen); S4, Oilseed (zhongshuang 11); S5, Oilseed (zhongyou 821); S6, Oilseed (BJ003); S7, Oilseed (BJ004).

The contents and components of GSL in the seeds were determined by performing an HPLC analysis. The total GSL contents did not show definite tendency among the seven resistant and seven susceptible plants. A high content and percentage of aliphatic GSL and a small percentage of indolic GSL and aromatic GSL were observed in the resistant plants, whereas the susceptible plants, such as cabbage and rapeseed, contained comparatively large amounts of indolic GSL and aromatic GSL (**Figure 4A**).

Although both resistant and susceptible plants contained a large amount of aliphatic GSL, the aliphatic GSL components differed. Progoitrin, epi-progoitrin, sinigrin and glucoraphanin greatly accumulated in the seeds of the resistant plants, whereas progoitrin, glucoraphanin, gluconapin and glucobrassicanapin were primarily observed in the susceptible plants (**Figure 4B**). In addition, relatively high levels of gluconasturtiin (aromatic GSL) and 4-hydroxyglucobrassicin (indolic GSL) were observed in the seeds of certain plants that were susceptible to *P. brassicae.*

### GSL in Roots During Disease Development

**FIGURE 5:**
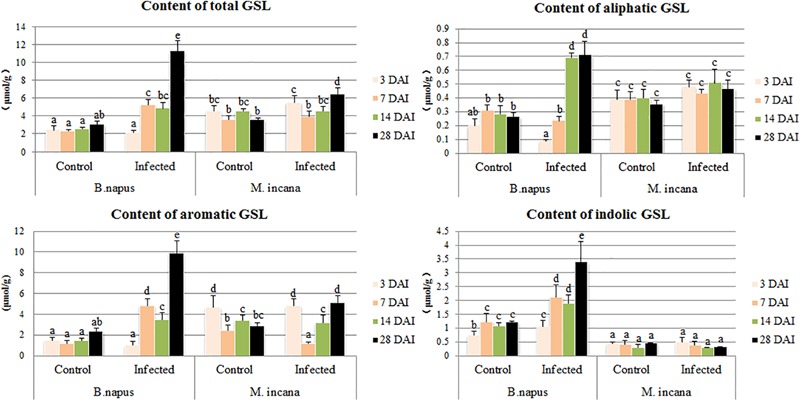
Content of glucosinolates (GSLs) in the roots of *B. napus* and *M. incana* at 3, 7, 14, and 28 days after inoculation (DAI). Error bars represent the standard errors of three independent experiments. Different letters above the bars indicate that the differences are significant (*P* < 0.05).

The GSL content in the roots of both resistant and susceptible plants was investigated at each time point after the *P. brassicae* inoculation (**Figure 5**). The content of GSL in the control roots of resistant plants was higher than that in susceptible plants. The pathogen inoculation greatly increased the total content of GSL in susceptible plants except at 3 DAI, whereas the total content of GSL was stable and increased only at 28 DAI in resistant plants (**Figure 3A**). By analyzing the various classes of GSL, distinct patterns of the various GSLs were observed between resistant and susceptible plants. The susceptible roots displayed uninterrupted increases in the synthesis of indolic GSL, and the content of indolic GSL was almost constant in resistant roots. Aliphatic GSL was significantly elevated at 14 and 28 DAI in the susceptible roots but was stable in resistant roots. Aromatic GSL was increased by *P. brassicae* inoculation in susceptible roots at 3 DAI, and an increase was observed only at 28 DAI in resistant roots.

**FIGURE 6:**
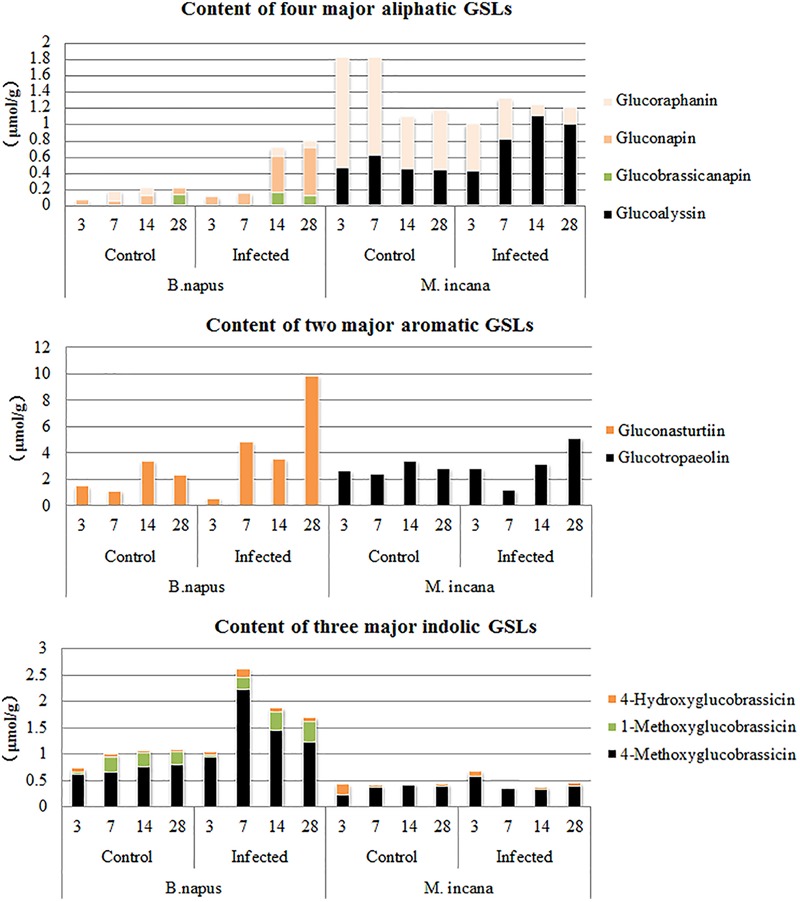
Major components of glucosinolates (GSLs) in the roots of *B. napus* and *M. incana* at 3, 7, 14, and 28 days after inoculation (DAI).

By examining the compositions of GSLs at various time points after inoculation, the compositions of GSLs were observed to greatly vary between resistant and susceptible plants (**Figure 6**). For example, of the two major aromatic GSLs, gluconasturtiin was present only in susceptible plants, whereas glucotropaeolin was mainly present in resistant plants at 28 DAI. The contents of the three major indolic GSLs were very low in resistant plants and did not change after inoculation; furthermore, only 4-methoxyglucobrassicin quickly increased at 3–7 DAI in susceptible plants. In contrast, the content of the aliphatic GSLs was relatively low in susceptible plants, and gluconapin exhibited increased accumulation at 14–28 DAI in susceptible plants, which did not exist in resistant plants. Two other aliphatic GSLs were detected in resistant plants, including glucoalyssin, which displayed increased accumulation during the secondary infection stage, and glucoraphanin, which displayed decreased content.

### Detection of JA Content and Induction of GSL by JA

**FIGURE 7:**
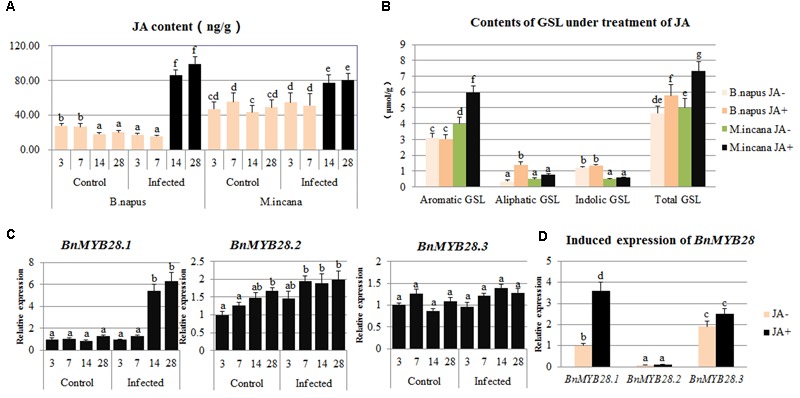
Contents of jasmonic acid (JA) in clubroot disease and induced content of glucosinolates (GSLs) and expression of *BnMYB28* by the exogenous JA treatment. **(A)** Contents of JA in clubroot disease. **(B)** Contents of GSL by exogenous JA treatment. **(C)** Expression of *BnMYB28* in clubroot disease. **(D)** Induced expression of *BnMYB28* by exogenous JA treatment. Error bars represent the standard errors of three independent experiments. Different letters above the bars indicate that the differences are significant (*P* < 0.05).

The content of endogenous JA was detected in the roots of *B. napus* and *M. incana*, both of which showed enhanced JA content during the secondary infection stage (**Figure 7A**). However, the JA level in *B. napus* was much higher than that in *M. incana*. After the exogenous treatment with 200 μmol/L JA, the total GSL content increased in both *B. napus* and *M. incana*. The content of aliphatic GSLs was significantly increased in susceptible plants, but only the content of aromatic GSL also increased in resistant plants (**Figure 7B**).

### Identification and Expression Pattern of *BnMYB28*

Three candidate *MYB28* genes were identified in *B. napus, B. rapa*, and *B. oleracea*. According to a multiple alignment of the 13 MYB28 proteins related to GSL biosynthesis pathways, these proteins are highly conserved, and two typical R2R3 MYB-DNA-binding domains exist in the N-terminal region (Supplementary Figure S1A). In contrast, the C-terminal region is highly polymorphic, resulting in the functional divergence of these genes. A phylogenetic tree was constructed and showed that all proteins could be divided into three subgroups and that BnMYB28.1 is more closely related to AtMYB28 than to BnMYB28.2 and BnMYB28.3 (Supplementary Figure S1B).

The expression patterns of the three *BnMYB28* genes after *P. brassicae* inoculation and exogenous JA treatment were analyzed by performing qPCR. The expression pattern of *BnMYB28.1* showed a significant increase during the secondary infection stage, whereas the expression of *BnMYB28.2* and *BnMYB28.3* remained almost constant (**Figure 7C**). Moreover, the treatment with exogenous JA greatly increased the expression of *BnMYB28.1* but not *BnMYB28.2* and *BnMYB28.3* (**Figure 7D**).

## Discussion

Clubroot disease has emerged as the main disease in Brassicaceae. Omics and molecular experiments have provided evidence regarding the involvement of GSL metabolites in clubroot disease. However, the exact role played by the metabolic pathway remains unclear. Therefore, an understanding of the regulation of GSL biosynthesis could provide useful information for studies investigating clubroot disease and other improvements in the value of agricultural crops.

### Primary Infection Occurred in Both Resistant and Susceptible Plants

First, different infection processes were observed, and the main differences between resistant and susceptible plants were determined. By dynamically monitoring the infection process, we observed that primary infection occurred in the root hairs of both resistant and susceptible plants, and the infection was correlated with the results of the PCR detection of the pathogen contents in the infected roots. Furthermore, even in particular non-host plant species, the germination of *P. brassicae* resting spores was stimulated, and the initial stage of *P. brassicae* pathogenesis was observed. These plant species, including soybean (*Glycine max*), leek (*Allium porrum*), winter rye (*Secale cereale*), and perennial ryegrass (*Lolium perenne*), may be useful in reducing inoculum levels in soil and increasing soil pH and can potentially be used as trap crops to reduce clubroot disease intensity (Ludwig-Muller et al., 1999a; Friberg et al., 2005, 2006; Bhattacharya and Dixon, 2010). Many studies have focused on the life cycle of *P. brassicae*. The present study indicated that both primary and secondary zoospores, which may essentially have the same identity, can cause both primary and secondary infections (Mithen and Magrath, 1992; Feng et al., 2013). Thus, an intriguing question is what is the role of primary infection in clubroot disease? It was still difficult to verify whether the primary infections were necessary during clubroot disease development. In particular, previous studies have focused on whether primary infection affects the initiation of resistance in host species or influences the development of a pathogen at the secondary stage (Kageyama and Asano, 2009; Feng et al., 2013).

### The Correlation of Clubroot Disease and GSL Contents in Seeds

Plants belonging to the Brassicaceae family are well known for their high GSL content. The total GSL contents in the seeds did not show definite tendency between resistant and susceptible plants in our study. In a previous report, the total seed GSL contents were suggested to be correlated with the susceptibility of Chinese cabbage varieties, and higher total GSL contents were found in eight different susceptible varieties than in two resistant varieties (Ludwig-Muller et al., 1997). Although our results are discrepant, our conclusion that susceptible plants contain a higher proportion of indolic and aromatic GSL is consistent. To improve the nutritional value of rapeseed oil and the quality of rapeseed meal, reducing the GSL content in the seeds is desirable; thus, double-low rapeseed has recently been bred and popularized (Nesi et al., 2008). In double-low rapeseed, only the content of aliphatic GSL is reduced, which results in enhanced proportions of indolic and aromatic GSLs. However, both the double-low rapeseed and double-high rapeseed strains tested in this study were susceptible, and double-low rapeseed had high proportions of indolic and aromatic GSLs. Thus, the higher content and proportion of indolic and aromatic GSLs are unlikely to result in susceptibility to clubroot. Furthermore, no significant difference was observed in the components and contents of GSLs between the roots and seeds in rapeseed, and it remains unknown whether changes in the GSL content in the seeds affect GSL content in other tissues (Kim and Ishii, 2006). Thus, whether the susceptibility of the main varieties to clubroot disease is correlated with low GSL levels in the seeds is unclear due to a lack of direct evidence.

### The Contents of GSL in Roots of Susceptible Plants

The contents and components of GSLs differ among plant genera and organs, suggesting that GSLs play diverse roles in metabolism in different organs and during various development stages (Ishida et al., 2014). Although the total contents of GSLs were relatively low in susceptible plants, all classes of GSL responded in susceptible plants at various time points after inoculation, suggesting that other GSLs, in addition to indolic GSL, play a role during disease development. The authors of a previous investigation of GSL content in four Chinese cabbages during clubroot disease development had the same conclusion (Ludwig-Muller et al., 1997).

To date, most studies have focused on the role of indolic GSL rather than aliphatic and aromatic GSLs in clubroot disease. Indolic GSL acts as a precursor for IAA production after *P. brassicae* inoculation, and low levels of root indole GSL may limit pathogen inoculation and development (Ludwig-Muller et al., 1999a; Ludwig-Muller, 2009). In this study, a correlation was observed between low contents of indolic GSL and resistance to clubroot. The content of indolic GSL increased in the *A. thaliana* ecotype Col, which is susceptible to clubroot disease (Ludwig-Muller et al., 1999b). In another study investigating the GSL content differences between resistant and susceptible Chinese cabbage, the two susceptible cultivars exhibited increased indolic GSL content at 14 and 20 DAI, whereas this content did not change in resistant cultivars (Ludwig-Muller et al., 1997). However, contradictory results have been reported. Two Arabidopsis mutants (*tu3* and *tu8*) both showed reduced disease symptoms, but only *tu8* exhibited decreased indolic GSL content compared to that in the wild type. Thus, similar to other disease responses, the disease symptoms caused by *P. brassicae* result from many regulation pathways, in addition to GSL, that interact to participate in the disease response. In our study, susceptible plants exhibited a significant increase in the indolic GSL content throughout the disease course. In addition, the elevation in indolic GSL is largely due to the accumulation of 4-methoxyglucobrassicin. Further study on the exact role of 4-methoxyglucobrassicin will provide useful information on clubroot disease. The earlier induction of indolic GSL may be responsible for the overproduction of IAA during the root hair infection stage. Furthermore, indolic GSL likely acts as a signaling molecule in the response to *P. brassicae*. However, the continuous induction during the secondary infection stage suggests that metabolites meet the needs of the obligate biotrophic pathogen. Thus, indolic GSL likely plays diverse roles in clubroot disease.

Knowledge regarding the aliphatic and aromatic GSLs in clubroot disease is limited. Aromatic GSL generally acts in the plant defense response against pests, whereas aliphatic GSL is a defense compound against plant pathogens. Initially, the induction of aliphatic GSL was hypothesized to be a defense response, but studies did not always support this hypothesis. The Arabidopsis mutant *gsm1-1*, which exhibits a reduced biosynthesis of aliphatic GSL, does not show improved resistance to clubroot disease (Tierens et al., 2001). In contrast, two susceptible Chinese cabbage varieties exhibited increased aliphatic GSL contents after *P. brassicae* inoculation, but no change was observed in the other two resistant varieties; this finding is consistent with our results because only the susceptible roots showed an increased content of aliphatic GSL during the late infection stage (Ludwig-Muller et al., 1997). The content of aliphatic GSL seemed almost unchanged in resistant plants, but not all the components remained constant. It showed the increased content of glucoalyssin, together with the decreased content of glucoraphanin, which made the role of aliphatic GSL more complicated in resistant plants. Gluconapin, which is present only in *B. napus*, was significantly increased during the secondary infection stage. Combined with the result that only seeds of susceptible plants contain gluconapin, gluconapin is likely a key factor in the pathogenesis of clubroot disease. Thus, particular components of GSLs, rather than the total contents of the GSLs, likely contribute to resistance or susceptibility.

The role of aromatic GSLs in clubroot disease is also uncertain, and the possible dual role of aromatic GSL during club formation is presented in non-Brassica species depending on the components of aromatic GSL (Ludwig-Muller, 2009). Both the resistant and susceptible plants showed enhanced content of aromatic GSL during the secondary infection stage, but the component was completely different (glucotropaeolin) in resistant plants, and gluconasturtiin was found in susceptible plants.

To summarize, these results agreed with the conclusion that disease severity is correlated with particular GSLs in one species, whereas the increase in other GSLs might be regarded as a defense response. Therefore, compared with the total content of GSL, the specific GSL may be more informative. Further studies investigating the exact role of 4-methoxyglucobrassicin and gluconapin should consider investigating hairy roots.

### JA May Be Responsible for the Induction of Aliphatic GSL in *B. napus*

Plant growth regulators always participate in clubroot disease as described in the introduction; this finding also suggested that JA plays a role during the secondary infection stage. In this study, both resistant and susceptible plants showed an enhanced accumulation of JA at 14–28 DAI, and this accumulation was even greater in susceptible plants; this finding is similar to previous results reported in Arabidopsis. Both Col-0 and Bur-0 showed increased JA accumulation after inoculation during the secondary infection stage, but the JA level and expression level of the JA-responsive genes were twofold to threefold higher in Col-0, suggesting that the JA response was activated (Lemarie et al., 2015). JA-dependent defenses generally act against necrotrophs, although *P. brassicae* is a biotroph (Bari and Jones, 2009). However, recent evidence has shown that some biotrophic pathogens trigger the defense response mediated by JA (Guerreiro et al., 2016). Interestingly, the JA response was activated during the secondary infection stage but not at the beginning after inoculation, suggesting that the JA-mediated defense response might begin during the cortex infection stage. This conclusion was also demonstrated in a study conducted by Lemarie et al. (2015).

Consistent results were obtained in several reports that showed high JA levels during the secondary infection stage in susceptible plants. The increased accumulation of JA in Col-0 was consistent with the induced expression of JA-responsive genes, suggesting that the JA signaling pathway is activated in clubroot (Siemens et al., 2006; Gravot et al., 2012). The jasmonate resistant 1 (*jar1*) mutant exhibited reduced JA-Ile accumulation and increased susceptibility to clubroot disease (Agarwal et al., 2011). Altogether, the exogenous JA treatment reduced the clubroot symptoms only in Col-0; thus, the JA response also participates in the weak defense in Col-0 (Lemarie et al., 2015). However, JA is upregulated in *B. rapa* roots during gall formation and can induce the content of indolic GSL and the activity of nitrilase (Grsic et al., 1999). This dual role of JA was deduced based on the interaction between susceptible hosts and *P. brassicae*. JA can activate a defense response against biotrophs, but it also participates in pathogenesis by mediating another pathway.

Some have shown the role of JA in GSL metabolism by exogenously applying JA, which increased the content of the indolic and aromatic GSLs in the leaves of *A. thaliana* (Guo et al., 2013), *B. juncea* (Augustine and Bisht, 2015), *B. napus* (Doughty et al., 1995), and various subspecies of *B. oleracea* (Kim and Juvik, 2011; Yi et al., 2016). The total GSL concentration increased up to 20-fold with the treatment of JA in *B. napus*, and the predominant components of the response were indolic GSLs, which together comprised 90% of the total GSLs in treated leaves (Doughty et al., 1995). However, knowledge regarding the role of JA in the modulation of GSL metabolism in roots is still limited. Our finding suggested a completely different result in the roots from that in the leaves, and it showed that exogenous JA treatments greatly increase the content of aliphatic GSL in *B. napus* and enhance the accumulation of aromatic GSL in *M. incana*. This result may be explained by the fact that the effect of JA on the GSL profile may differ among organs. In considering the regulatory role of JA, it can be deduced that JA-induced aromatic GSL may be involved in the defense against *P. brassicae*, whereas aliphatic GSL induced by JA in *B. napus* may participate in pathogenesis.

### *BnMYB28* May Regulate Clubroot Disease by Modulating JA-Mediated Aliphatic GSL Metabolism

*Brassica napus* is an allotetraploid derived from the interspecific hybridization of *B. rapa* and *B. oleracea*. Due to the occurrence of polyploidy and genome-wide rearrangements, the regulation of aliphatic GSL biosynthesis in Brassica species is expected to be highly complex compared to that in the closely related diploid Arabidopsis (Qu et al., 2015). *AtMYB28* has been found to positively regulate the synthesis of aliphatic GSLs (Li et al., 2013). Three paralogous *MYB28* genes have been identified in *B. napus, B. rapa*, and *B. oleracea*; compared to Arabidopsis, these *MYB28* genes have a high level of sequence homology. A high collinearity has been observed among these genes in *B. napus, B. rapa*, and *B. oleracea*. The existence of multiple paralog genes in allopolyploid plants suggests that highly diverse expression patterns and functions exist among the paralogs of the candidate genes (Seo et al., 2016). The overexpression of the three paralogous *BrMYB28* genes in transgenic Chinese cabbage resulted in enhanced content of total GSL in all T_1_ and T_2_ transgenic plants. Compared to non-transgenic plants, overexpression of *BrMYB28.1* resulted in the highest total GSL contents and increased content of the aliphatic, indolic, and aromatic GSLs (Seo et al., 2016). In studies overexpressing *BoMYB28* in Chinese Kale (*B. oleracea*), aliphatic GSL contents were higher in the overexpression lines than those in non-transgenic plants, and enhanced expression levels of aliphatic GSL biosynthesis-related genes were observed (Yin et al., 2017). Thus, the regulatory mechanism of GSL biosynthesis in Brassica crops differs from that in *A. thaliana*. Although a functional validation of the candidate *MYB28* genes has been performed in *B. rapa* and *B. oleracea*, knowledge regarding the functions of these genes remains limited, particularly in clubroot disease. Among the three *BnMYB28* candidates, the expression level of *BnMYB28.2* was extremely low in the root; this finding is consistent with results showing that *BnMYB28.2* is associated with the seed GSL content *B. napus* (Qu et al., 2015). *BnMYB28.1*, which showed the highest similarity of amino acid sequence with *AtMYB28*, was induced by *P. brassicae* inoculation during the secondary infection stage and exogenous JA treatment. Thus, the increased content of JA during the secondary infection stage may induce the expression of *BnMYB28.1*, which caused the accumulation of aliphatic GSL in clubroot disease development. However, this assumption requires further investigation.

In summary, GSL metabolites are more likely to play a role during disease development than to act as defense compounds in clubroot. However, the possibility that GSL metabolites participate in the defense response through degradation cannot be eliminated. We hypothesize that a dual role of JA was suggested to participate in clubroot disease via different regulation methods. Further studies exploring the exact role of 4-methoxyglucobrassicin and gluconapin in hairy roots should be performed using *in vitro* culture. The *MYB28* TF, which regulates clubroot disease development by modulating JA-mediated aliphatic GSL metabolism, may play a specific role in clubroot disease development. Future studies investigating the function of *BnMYB28.1* are fundamentally important to better understand the complex mechanisms controlling GSL accumulation during clubroot disease development. Understanding GSL metabolism and the regulatory network in rapeseed clubroot could enable the rational engineering of GSL content to boost plant protection without compromising the crop quality.

## Author Contributions

LX and XF designed the study. LX, HY, LR, WC, LL, LZ, and RY performed the experiment and analyzed the data. LX wrote the paper. XF revised the paper. All authors read and approved the final manuscript.

## Conflict of Interest Statement

The authors declare that the research was conducted in the absence of any commercial or financial relationships that could be construed as a potential conflict of interest.
