# Supplementary material for: Jasmonic Acid-Mediated Aliphatic Glucosinolate Metabolism Is Involved in Clubroot Disease Development in Brassica napus L
Source: Front Plant Sci. 2018 Jun 4;9:750. doi: 10.3389/fpls.2018.00750 (PMC5996939; doi:10.3389/fpls.2018.00750)
Supplement: Supplementary file 1 [file Table_1.DOCX]

**Supplementary material**

Table S1 Primers for PCR amplification of *P. brassicae*.

| **Name** | **GenBank No.** | **Primer sequence** |
| --- | --- | --- |
| *BnACT2* | GQ339782.1 | F: CTCTTCCACATGCCATCCTTC |
|  |  | R: CTCTTCCACATGCCATCCTTC |
| *Mi18S* | KF022689.1 | F: CAGAACGACCAGCGAACCAA |
|  |  | R: CGATGCGAGAGCCGAGATAT |
| *PbTC1* | AF231027.1 | F: GTGGTCGAACTTCATTAAATTTG |
|  |  | R: TTCACCTACGGAACGTATATGT |
