# Supplementary material for: Jasmonic Acid-Mediated Aliphatic Glucosinolate Metabolism Is Involved in Clubroot Disease Development in Brassica napus L
Source: Front Plant Sci. 2018 Jun 4;9:750. doi: 10.3389/fpls.2018.00750 (PMC5996939; doi:10.3389/fpls.2018.00750)
Supplement: Supplementary file 2 [file Table_2.DOCX]

**Supplementary material**

Table S2 Primers for qPCR.

| **Name** | **Ensemblplant** | **Primer sequence** |
| --- | --- | --- |
| *BnMYB28.1* | BnaA03g40190D | F: TTGCGGCTAAGGTCACTTCT |
|  |  | R: CTCCTCGTTGTGGTCATCTCT |
| *BnMYB28.2* | BnaC09g05300D | F: AGACGCTCATCTCTTACATTCAC |
|  |  | R: TCTCATTGTCGGTTCTTCTAGGT |
| *BnMYB28.3* | BnaCnng43220D | F: GAGCAGATTCGCAATGAAGAGG |
|  |  | R: GGACACATCGGACATAAGAAG |
