# Supplementary figures and images for: Jasmonic Acid-Mediated Aliphatic Glucosinolate Metabolism Is Involved in Clubroot Disease Development in Brassica napus L
Source: Front Plant Sci. 2018 Jun 4;9:750. doi: 10.3389/fpls.2018.00750 (PMC5996939; doi:10.3389/fpls.2018.00750)

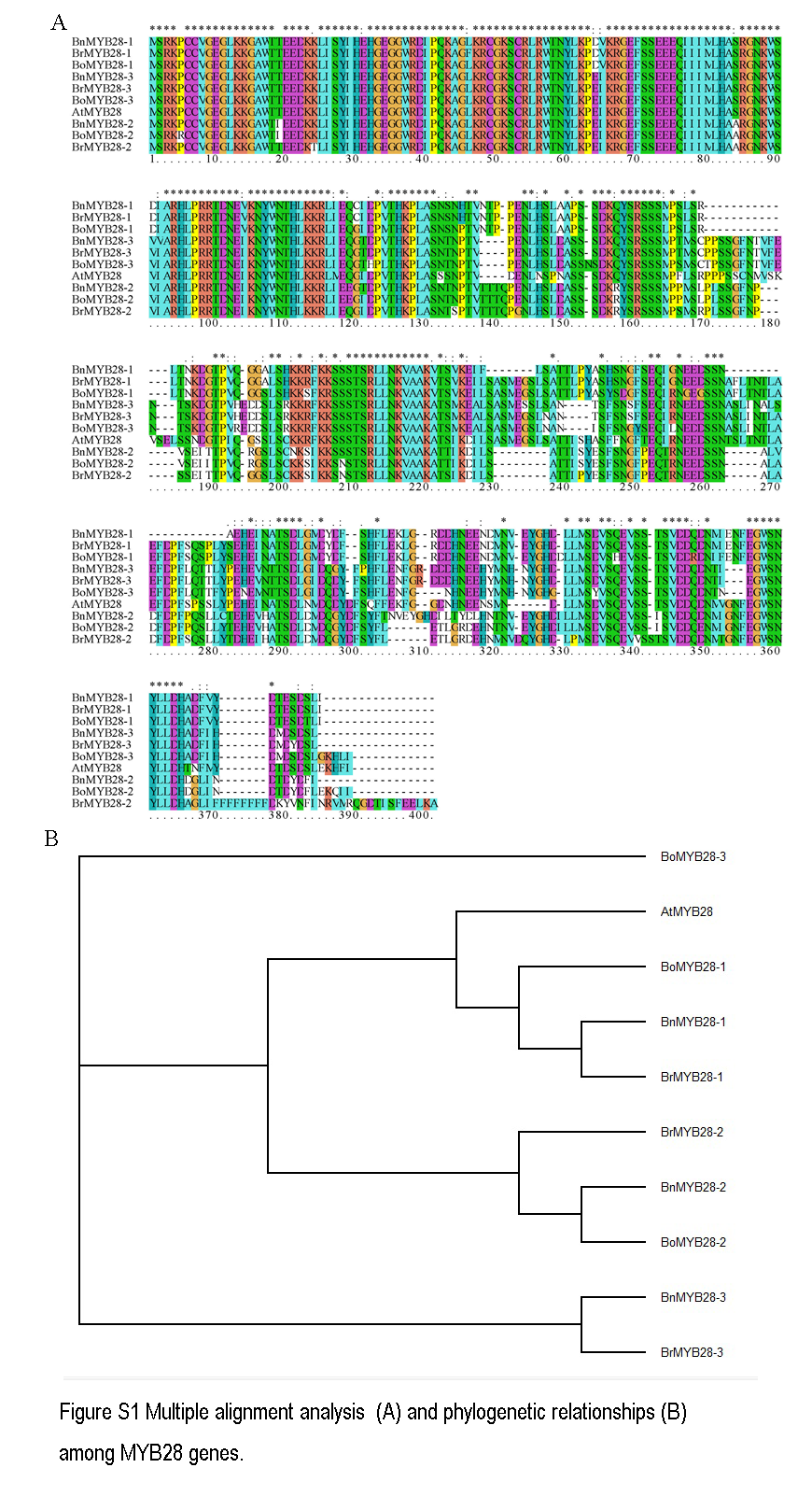

Supplement: Supplementary file 3 [file Image_1.tif]
